# Tracking the career development of scientists in low- and middle-income countries trained through TDR’s research capacity strengthening programmes: Learning from monitoring and impact evaluation

**DOI:** 10.1371/journal.pntd.0006112

**Published:** 2017-12-07

**Authors:** Béatrice Halpaap, Mahnaz Vahedi, Edith Certain, Tini Alvarado, Caroline Saint Martin, Corinne Merle, Michael Mihut, Pascal Launois

**Affiliations:** 1 UNICEF/UNDP/World Bank/WHO Special Programme for Research and Training in Tropical Diseases (TDR), World Health Organization, Geneva, Switzerland; 2 Consultant, TDR, World Health Organization, Geneva, Switzerland; 3 Centre Hospitalier Universitaire de Reims, Pôle Recherche et Santé publique, Unité d'aide méthodologique, Reims, France; London School of Hygiene and Tropical Medicine, UNITED KINGDOM

## Abstract

The Special Programme for Research and Training in Tropical Diseases (TDR) co-sponsored by UNICEF, UNDP, World Bank and WHO has been supporting research capacity strengthening in low- and middle-income countries for over 40 years. In order to assess and continuously optimize its capacity strengthening approaches, an evaluation of the influence of TDR training grants on research career development was undertaken. The assessment was part of a larger evaluation conducted by the European Science Foundation. A comprehensive survey questionnaire was developed and sent to a group of 117 trainees supported by TDR who had completed their degree (masters or PhD) between 2000 and 2012; of these, seventy seven (77) responded. Most of the respondents (80%) rated TDR support as a very important factor that influenced their professional career achievements. The “brain drain” phenomenon towards high-income countries was particularly low amongst TDR grantees: the rate of return to their region of origin upon completion of their degree was 96%. A vast majority of respondents are still working in research (89%), with 81% of respondents having participated in multidisciplinary research activities; women engaged in multidisciplinary collaboration to a higher extent than men. However, only a minority of all have engaged in intersectoral collaboration, an aspect that would require further study. The post-degree career choices made by the respondents were strongly influenced by academic considerations. At the time of the survey, 92% of all respondents hold full-time positions, mainly in the public sector. Almost 25% of the respondents reported that they had influenced policy and practice changes. Some of the challenges and opportunities faced by trainees at various stages of their research career have been identified. Modalities to overcome these will require further investigation. The survey evidenced how TDR’s research capacity grant programmes made a difference on researchers’ career development and on south-south collaborations, by strengthening and localizing research capacity in lower income regions, and also showed there is more that needs to be done. The factors involved, challenges and lessons learnt may help donors and policy makers improve their future interventions with regard to designing capacity strengthening programmes and setting funding priorities.

## Author summary

The Special Programme for Research and Training in Tropical Diseases (TDR) co-sponsored by UNICEF, UNDP, World Bank and WHO has been providing training grants to strengthen research capacity in low- and middle-income countries for over 40 years. In order to assess to what extent TDR’s grants made a difference on the career development of these grantees, a survey tool was developed and implemented in collaboration with the European Science Foundation. The survey was answered by 77 individual trainees who completed their degree (masters or PhD) with support from TDR between 2000 and 2012. The study provided valuable information on factors involved in the career development of the trainees and influencing the local retention of the capacity that has been built, to prevent “brain drain”. Encouraging aspects, such as a 96% of the capacity being retained locally, a 92% full-time employment rate at the time of the survey, or 89% of the respondents still working in research showed the positive influence of TDR’s capacity strengthening grants on researchers’ career development. This was in line with 80% of the respondents rating TDR’s support as “very important”. The challenges, lessons learnt and further opportunities identified may be helpful to donors and policy-makers when designing research capacity programmes, fostering south-south collaboration, and setting funding priorities.

## Introduction

The Special Programme for Research and Training in Tropical Diseases (TDR), co-sponsored by UNICEF, UNDP, the World Bank and WHO, has a long track record in research capacity strengthening. Created in 1975 to support research and research capacity strengthening in the fight against tropical diseases, TDR’s goal is to improve health and reduce the burden of infectious diseases in low- and middle-income countries (LMICs). For more than 40 years, TDR has strengthened health research capacities in these countries by: i) supporting individuals’ education and training through fellowships, scholarships and learning-by-doing programmes for specific skills, particularly on good practice for health research and fostering mentorships; ii) supporting institutional capacity by establishing national and international training and research centres; and iii) developing networks and collaborative research projects [1].

Regular external reviews of its research capacity strengthening programmes have helped TDR to evolve its strategy in light of the global environment so as to remain a fit-for-purpose programme. The latest evaluation of TDR’s contribution to career development of a selected group of individuals and institutional capacity development grantees was conducted in 2010. The main objective was to identify factors that positively influenced and improved the research capacity and career development of TDR trainees and that are of broader relevance to the objectives and goals of international development and aid agencies [2, 3]. One of the recommendations was to better track the career development of grantees to help evaluate the influence of these early learning supports.

To respond to these recommendations a career tracking survey tool was developed to study the potential links between the grants received by TDR trainees and their career development. The survey is conducted every 2–3 years to provide quantitative and qualitative data to better understand TDR’s grants impact on grantees’ careers. It provides an instant view of a trainee’s career, with performance indicators to allow monitoring and evaluation of career development. This survey tool was developed and implemented in collaboration with the European Science Foundation in France, a European structure that generates evidence to support the decision-making of countries or organizations. It was implemented in 2014 to study the contribution of TDR support on TDR training grantees’ careers between 2000 and 2012. The survey responses have highlighted the challenges, bottlenecks and opportunities of different research career stages, which are being used to identify intervention points or specific actions needed to achieve desirable career progression.

## Methodology

### Joining the European Science Foundation study to synergize efforts

TDR was invited to respond to a call for research support and funding organizations to join a doctorate career tracking project. The survey was launched in late 2014 by the European Science Foundation in Strasbourg, France. The aim of this call was to develop a methodology to design and implement a career tracking survey tool.

Five organizations joined the study: the AXA Research Fund, Paris, France (AXA); the Fonds National de la Recherche, Luxembourg (FNR); the Goethe Graduate Academy (GRADE), Frankfurt, Germany; the Paul Scherrer Institute (PSI), Villingen, Switzerland and TDR. All data were disaggregated by organizations. Six hundred and thirty eight (638) trainees from the five partners responded to the survey with the following breakdown: 110 from the AXA fund, 84 from FNR, 105 from GRADE; 133 from PSI and 77 from TDR. The aggregated results from the 638 participants have been published [4].

Data were then disaggregated in 2015 and results specific to the TDR trainees are presented in this paper.

### Survey populations

A total of 304 TDR trainees who completed their doctorate or master’s degree between 2000 and 2012 with a TDR grant were identified in the TDR information and management system. These included recipients of any of the following scheme of grants: research training grants (RTG); re-entry grants (REG); the Multilateral Initiative on Malaria (MIM); research grants and institution strengthening grants (ISG). RTGs were awarded to individuals in LMICs to pursue studies leading to a postgraduate degree (MSc or PhD) at their home country institution, in another LMIC or in a high income country. REGs were intended to facilitate the career development of young scientists returning to their home institution within 12 to 24 months, following completion of a graduate degree (MSc or PhD) or a post-doctoral fellowship. ISGs were designed to provide up to three years of support to an institution or research group to enhance infrastructure and the research environment. MIM grants [3] were used to provide support to core African research groups for the development of malaria control tools (Box 1).

**Box 1: Definition of the various TDR grants included in the survey**

**RTG**: **Research training grants** were awarded to individuals from low- and middle-income countries working in a research institution in order to pursue studies leading to the acquisition of a postgraduate degree (MSc, PhD), or for acquiring specialized skills (short-term courses, post-doctorate). The training took place in the individuals’ own country, in another low- or middle-income country or in a high-income country. RTGs were generally allocated for a specific period which do not always cover the full time of a degree: from a couple of weeks to two or three months for a short-term course, for one to two years for an MSc degree, for three to four years and exceptionally up to five years for a PhD degree, and for a couple of months to one year for a post-doctorate degree.

**REG**: **Re-entry grants** were intended to facilitate the career development of young scientists returning to their home institution within 12 to 24 months following completion of a graduate degree (MSc, PhD) or post-doctoral training period. Re-entry grants were awarded on a competitive basis for a three-year period.

**MIM: The Multilateral Initiative on Malaria research grants** provided support to core African research groups for the development of malaria control tools. The aim was to promote partnerships, collaboration, technology transfer and training opportunities by supporting large, multi-country collaborative research projects and networks in malaria endemic countries. Financial support could be for an initial period of one to three years, subject to annual review and satisfactory progress. Long-term support was considered on a case-by-case basis.

**ISG**: **Institution strengthening grants** were intended to provide long-term support to institution or research group development programmes. The objectives of the grant were to: (i) promote the development of the infrastructure and research environment; (ii) improve training opportunities, scientific expertise in biomedical and social sciences and information and communications systems; and (iii) foster opportunities for scientific collaborations. Financial support could be for an initial period of one to three years, subject to annual review and satisfactory progress. Long-term support was considered on a case-by-case basis.

Information on all individuals and institutions that received grants between 2000 and 2012 was extracted from the TDR information management system and tabulated for range and scope of research topics.

Trainees were contacted individually, through e-mail, to ascertain their willingness to participate in the career tracking survey and to update their personal information. From a total of 304 trainees identified, 117 trainees (39%) responded positively while 187 did not respond, either due to out of date e-mail addresses or possible lack of interest.

### Designing the survey: Building upon existing questionnaires from various institutions

The questionnaire design was based on existing surveys of doctorate graduates conducted by the Organization for Economic Cooperation and Development (OECD), Eurostat, the European Commission Marie Sklodowska-Curie actions, Wellcome Trust, UNESCO and the US National Science Foundation. The range of topics covered by the survey included demographics, mobility (virtual, physical and sectoral), research outcomes, roles and responsibilities, competence development and skills utilization. Several drafts of the questionnaire were reviewed by the five participating organizations and pre-tested in-house by ESF staff members, with the final questionnaire peer-reviewed by two independent international experts. The resulting questionnaire contained 52 questions, written in English.

### Protecting data

Participants were informed about the detailed data protection and confidentiality arrangements that were in place for the survey such as the anonymization of replies before analysis. This included destroying all contact details before conducting any survey analysis and avoidance of any questions likely to collect sensitive or identifying information of any kind (date of birth, thesis title, disciplinary field, institution name, etc.). Written assurance was also given that contact details would only be used for the purpose of contacting the trainees during the data collection phase. Since ESF is located in Strasbourg, France, the modalities of the survey were declared to the Commission Nationale de l’Informatique et des Libertés (CNIL), the independent French authority protecting privacy and personal data.

### Launching the survey, following up with participants and preparing for statistical analysis

The list of TDR trainees and their contact details were shared with ESF and names and e-mail addresses entered into an online survey database. The survey was launched with an explanatory cover note from ESF in September 2014. The questionnaire and an introductory message were sent to each of the 117 participants.

Any queries received by the ESF team from participants were dealt with on an individual basis, including practical questions regarding completion of the questionnaire. The number of respondents was logged on a daily basis and the percentage of responses on a weekly basis. A total of five reminders to participate in the survey were sent. The survey was closed in November 2014 and all respondents were thanked for their participation.

The survey data were imported into the Statistical Package for the Social Sciences (SPSS) for analysis by ESF.

## Results

### Respondent profiles: Illustration of TDR training grant focus

Among the 304 TDR trainees identified, 117 trainees expressed availability to participate and were included in the survey. These included 54 RTG, 29 REG, 18 MIM and 16 ISG grants. Ultimately, 77 trainees responded to the survey (66% of those included). Unfortunately it was not possible to break down the analysis by grant as 68% responded to the question *“Do you know the type of grant you received from TDR?”* with *“don’t know”* and the survey was anonymous.

**Fig 1:**
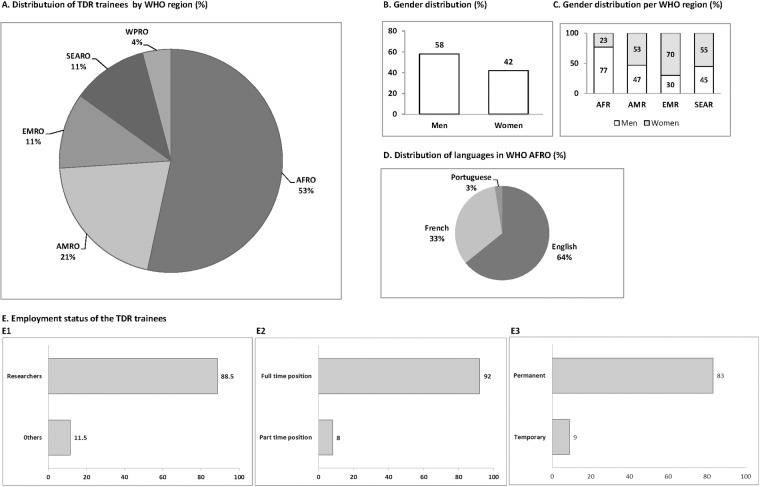
Profile of the 77 TDR trainees who responded to the survey. (A) geographic distribution by WHO region (AFR for African region; AMR for region of the Americas, SEAR for South East Asia region, EMR for Eastern Mediterranean region and WPR for Western Pacific region); (B, C) gender distribution in total and by WHO region, respectively; (D) use of language in AFR; (E) employment status of trainees by function (E1), and by nature (E2 and E3).

WHO Member States are grouped into six regions: Africa (AFR), the Americas (AMR), South-East Asia (SEAR), Europe (EUR), Eastern Mediterranean (EMR), and Western Pacific (WPR). Profiles of the TDR trainees who responded to the survey are shown in Fig 1A. The majority of respondents came from AFR (53%); 21% originated from AMR, mainly from Brazil (59%) and Argentina (25%); 11% were from SEAR, 11% from EMR and 4% from WPR.

Of the 77 respondents, 58% were men and 42% were women (Fig 1B). Representation of women was slightly higher than in the group of 304 initially contacted (62% men, 38% women). As shown in Fig 1C, women are well represented in all WHO regions except AFR where men are more represented (77%) than women (23%). In EMR, women are more represented (70%) than men (30%).

In terms of age, the majority of respondents were between 35 and 45 (51%). Women were slightly older than men: 34% of women were above 50 years of age as compared to 18% of men. In all WHO regions, except AMR, the vast majority of women have children (92%) but only 29% of women had children in AMR. Further investigation would be needed to understand any potential barrier for AMR women with children to access TDR training grants. Fifty-eight percent of men and 42% of women had other caring responsibilities such as care of an elderly person or an adult with a disability.

In AFR, the majority of respondents were from English speaking countries (64%) followed by French (33%) and Portuguese (3%) (Fig 1D). The response rate (number of trainees who responded to the questionnaire / number of trainees who received the questionnaire) was higher in Francophone (87%) than in Anglophone (55%) trainee sub-groups. When aiming at enhancing support to Francophone and Lusophone countries, further study may be needed to better understand the factors involved.

### The influence of type and length of the support received

The 77 respondents were supported by TDR to obtain either a MSc (14 respondents), a medical doctorate (MD) (5 respondents) or a PhD (58 respondents).

All trainees studying for a master’s degree obtained their degree through structured means, involving a combination of defined courses and independent research. For trainees studying for a PhD, the majority of respondents (86%) achieved their degree through the traditional means of an independent research study under the guidance of a supervisor and only 14% through structured means. There was no relationship between the time taken to complete the degree and the structure followed. There was also no difference in the time taken and the structure followed to complete their degree between men and women.

The median time taken by respondents to complete their PhD was four years. Support provided by TDR did not always cover the full duration of the degree and ranged from one year (28%) or less (6%) to two years (20%), three years (25%), or more (21%). More men were supported for three years (30%) as compared to women (19%), and more women were supported for one year or less (39%) as compared to men (30%). The reasons for the variation of length of support are not clear. It would be important to better understand why duration of support was shorter for women than for men and what the implications were. Indeed early career support to acquire a degree is known to be a key factor for career development and a potential future leadership role [5].

Eight respondents (seven men from AFR and one woman from AMR) took a career break for one year or more. Of the seven men, only one found it very difficult to return to their previous position. The only woman who took a study break found it relatively easy to return to her position. However, the reasons for having taken a study break were not clearly explained; a more explicit question will be added in the next survey.

### Creating opportunities for geographical mobility

A proportion of respondents (35% overall) moved outside of their country of origin to complete their degree, the majority from AFR. Forty-one percent of AFR respondents who moved abroad went to high-income countries, mainly in North America and Europe.

Overall, 65% of respondents completed their degree in their region of origin. Seventy-nine percent of TDR grantees from AFR who completed their degree in their region of origin were trained in three countries: Kenya, Nigeria and South Africa. Sixty-nine percent of TDR grantees from AMR who completed their degree in their region of origin were trained in Argentina and Brazil. All of the countries where training took place have a relatively high national gross domestic income, with a well-developed health research structure and capabilities [6]. Thus, the survey showed the great benefit of TDR and other agencies supporting capacity strengthening programmes to promote collaboration between scientists in countries with more advanced health research capacities and countries with lower health research capacities within the same region.

The gain of south-south collaboration as compared to north-south collaboration in term of career development was analysed based on the 41 trainees from AFR. Fifteen (15) studied in high-income countries (north-south collaboration), 20 studied in three other African countries (Kenya, Nigeria and South Africa) (south-south collaboration) while six studied in their own country. There was no difference in response to the different questions between trainees who studied in high-income countries and those who studied regionally. This may suggest that south-south collaborations are as effective and at a lower cost than north-south collaborations.

### Career development following TDR support

#### Returning to country of origin and avoiding “brain drain”

Almost all respondents—regardless of where they originated—returned to their region of origin following completion of their studies, with only 4% choosing to stay in a high-income country. The study found no difference between genders. This indicates that TDR support did not lead to brain drain from low- and middle-income countries to high-income countries, which is one of the primary concerns of any grant funding development programme. One of the factors contributing to the significant return rate may be the framework and strict criteria used for the selection of trainees. Indeed, trainee candidates are required: (i) to have been employed for the previous 12 months or longer in an organization registered in an LMIC; and (ii) to have a contract with the same organization confirming that they will be given the same (or equivalent) position following completion of their training.

### Fostering career development in research

All of the TDR trainees who responded to this survey were employed, with 95% holding a position at a university or research institution and 89% working as academic researchers.

Fig 1E provides details of TDR trainees’ current employment. Most of the respondents held a full-time position with more than 30 hours per week (92%) in either a permanent (83%) or temporary position (9%). Women were more often in permanent full time positions (91%) than men (78%). While the number is small, only men were self-employed (2%).

The vast majority of respondents worked in the public sector (83%) in non-profit (79%) or for-profit (4%) institutions, followed by the private sector (12%) and others, including public-private partnerships (2%). Twenty eight (28%) were directly funded by their employer, while 72% were employed on grants funded by some other external party.

Table 1 presents TDR respondents working as academic researchers (89%) by career stages as per the Frascati definition [7, 8]. The only difference between men and women was that a higher proportion of women described themselves as R1 researchers (first stage) and more often they held positions as junior researchers. This was the case for all WHO regions.

**Table 1:** TDR respondents by career stage (Frascati definition) [7, 8] and gender (in %).

Career stages	Total	Men	Women
**R1 First stage researcher**	17	14	21
**R2 Recognised Researcher**	17	17	17
**R3 Established Researcher**	27	26	29
**R4 Leading Researcher**	36	37	33
**Others**	3	6	0

The minority not working as researchers (11%) were asked to indicate the reason(s) for this. The most common reasons cited were the difficulty of obtaining a suitable academic research position (100%), the difficulty to secure a tenured post (100%), the lack of research career opportunities (80%) and the low remuneration in research positions (75%).

In terms of occupational areas, the highest proportion of respondents worked in life sciences (47%), followed by education (34%), training (31%), healthcare (31%) that included healthcare practitioners and healthcare support occupations, social sciences (5%) and administrative support.

Table 2 clearly shows that a higher proportion of men were involved in management. However, similar proportions of women and men worked in life sciences, education, healthcare, social sciences and administrative support. There was no difference based on country of origin, country of study, country of work and their career stages.

**Table 2:** Occupational area and gender of TDR respondents (in %) (percentages do not add up to 100% since respondents can work in more than one occupational area).

Occupational areas	Total	Men	Women
**Management**	17	22	9
**Life sciences**	45	44	47
**Social sciences**	6	4	5
**Education and training**	32	31	34
**Heath care**	31	31	31
**Administrative support**	5	4.5	6

In general, it is quite difficult to compare salaries across the geographical spread of the various WHO regions. However, some gender differences in salary levels were evident, since a higher proportion of women earned less than €20 000 per year (55% women versus 44% men) regardless of the region of origin. This is perhaps due to the fact that women more often held positions of junior researchers (Table 1). It could also reflect the worldwide issue of the gender pay gap.

### Enhancing regional and international collaborations

The survey asked respondents to indicate in how many different countries they had physically studied or worked for a period of more than three months during and after TDR support (physical mobility). The majority of respondents had studied and worked solely in their own country (72%) while 28% percent had studied or worked in other countries. It is worth noting that international physical mobility was higher for AFR respondents (48%). In general the survey showed a strong mobility to countries with more advanced health research capacities. The highest international physical mobility was to Europe (60%) and North America (38%) then Argentina and Brazil (11% each) and Australia (7%).

Virtual mobility, or collaboration via information and communication technology platforms, was also considered. The majority of respondents (75%) acknowledged virtual mobility had taken place solely within their own countries. From the 25% remaining respondents, the highest international virtual mobility was to countries in Europe (46%) and North America (31%). As was the case with physical mobility, virtual mobility was higher for AFR respondents (42%). Interestingly, international physical mobility to Europe was higher than virtual mobility.

The survey showed that 58% of respondents conducted research in collaboration with researchers based in another country, mainly in Africa, Europe and North America, through a joint publication (55%) and/or a joint project (52%), in line with the mobility trends described above.

### Stimulating multidisciplinary and intersectoral collaborations

There was a considerable proportion of respondents who reported having engaged in multidisciplinary research activities (81%). Multidisciplinary collaboration was reflected through either joint publications (81%), collaborating at distance with occasional face-to-face (67%) or through web-based technologies (52%). Women seemed to engage in multidisciplinary approaches to a higher extent than men. Indeed, a higher proportion of women worked with researchers from a different field of expertise, either through joint publications (89% for women versus 74% for men) or virtual collaborations (63% for women versus 44% for men). This could be due to the fact that more women work in the field of social sciences than men. In a previous study analysing TDR support of 116 research training grants, 11/36 (30.50%) women and 11/80 (13.75%) men worked in the domain of social sciences. However in a recent study analazing gender differences in scientific collaborations, it is clear that women in the natural sciences domain have more collaboration in other fields than men [9]. Further research would be needed to better understand why TDR women trainees tend to engage in multidisciplinary approaches more often than men.

Intersectoral collaboration, in terms of joint activities between research institutions, industry or commercial ventures, was limited: 23% worked on a joint publication and 19% collaborated on a joint research project with industry. Men collaborated only slightly more frequently with industry (33%) than women (26%). There is a clear need to encourage intersectoral collaboration. This could be done though promoting and fostering mobility between research institutions, government and nongovernmental agencies, and the public and private sectors. It would help to make the career perspective after graduation more attractive and to reduce existing barriers to collaborative work between these sectors. Some of these barriers to career development are attitudinal, reflecting a lack of knowledge and sometimes a negative perception that academic staff may have about a career outside the university’s walls. The quality of career mentorship provided at the doctoral level could be an essential element to help overcome these concerns. Other barriers could be structural and institutional, bringing into question the reliance on publication output as the sole or main criterion for scientific recognition and career development.

### Making a change: Impact on trainees’ current activities

Respondents, regardless of the region they came from, reported that they regularly used their doctoral skills in their current position (92%). They most often used these skills in managing research activities (74% of respondents dedicated more than 20% of their time to these activities). This was followed by staff management activities (47%), which included supervising students either at undergraduate and master levels (82%) and/or PhD level (65%) or supervising their peers’ work (75%); teaching activities (46% dedicated more than 20% of their time) and administrative activities (37% dedicated more than 20% of their time). Some dedicated time to transferring technology to industry (21%). There was no significant difference between genders in any of these activities.

Respondents reported having made presentations at national (73%) and international conferences (72%) and women were more active than men in international presentations (75% for women versus 66% for men).

Over 70% of respondents had been either lead authors (65%) or co-authors (70%) on peer-reviewed publications in the last 12 months. Similar proportions of men and women had been lead authors (69% for women versus 62% for men) but a higher proportion of men were co-authors (55% for men versus 45% for women) on peer-reviewed publications.

In terms of research and development, 20% of respondents had produced new research software resources and 9% of them had filled a patent. None of them had registered or licenced a product in the last 12 months.

Almost 25% of respondents claimed that their work had made a significant impact on influencing changes in policy and practice. This relatively low percentage could be due to the fact that respondents come from a largely academia-based group (98% held a position in university or in research institutions and 89% worked as academic researchers) which usually report impact more through publications, conference presentations and research awards. All trainee contact details or other identifying information of any kind (date of birth, thesis title, disciplinary field, institutional name, etc.) were destroyed before conducting any analysis. As a consequence, there was no possibility to verify if the work of the trainee had an impact on policy and practice. However, this percentage suggests the need to maintain and enhance efforts to bridge the gap between health research and policy-making and practice, as well as the need to capture such evidence in a systematic way. Indeed, the lack of evidence on translating research results into health policies, interventions or new tools has been identified for decades as a weakness in the evaluation of research capacity strengthening organizations [10].

Activities to communicate results to the public had been undertaken by 30% of respondents and media coverage was achieved by 22% of respondents. Men were more likely than women to claim impact on policy and practice changes (29% versus 19%), to communicate to the public (40% versus 16%) and to receive media coverage (27% versus 16%).

### Degree, post degree, choice of career and TDR support

Respondents were asked to rate the importance of TDR support on achieving their professional career goals. Eighty (80) percent rated TDR support as very important, and 91% of respondents rated the TDR support as very or fairly important; no difference was observed between genders. This outcome is substantially higher when compared to the other four organizations involved in this survey, which scored an average of 54% of importance with the support received. These results confirm the need for research capacity strengthening in low-and middle-income countries and the catalytic role that TDR has played in research career development. Two additional elements, the first post-doctorate employer and the academic advisor, were rated as important for career progression by 64% and 63% of the respondents, respectively.

The post-degree career choices made by the respondents were strongly influenced by academic considerations. The most important reason influencing the decision to accept a post-doctorate position was the willingness to get additional training in the same area of their degree (70%). This was seen as a necessary step towards the employment they aspired to (67%). This is an important result to be taken into consideration when implementing future research capacity strengthening programmes for development. Increasingly, countries have identified the need for building capacity in research for implementation in order to enhance health care delivery and reach vulnerable populations. Research for implementation helps solve implementation bottlenecks, identify optimal approaches for real life settings and speed up the bench-to-bedside translation. TDR has recently shifted its strategic focus toward research for implementation, and is building upon capacity already developed with previous trainees.

## Discussion

The survey generated valuable information that highlighted the positive impact of TDR training grants on the research career development of its trainees. The response rate (68% of all TDR trainees contacted) was high in comparison to average online surveys (30%) [11].

However, this study presents two main limitations. Fist the population of TDR grantees who responded is small, i.e. 77 respondents from the 117 TDR trainees who had a valid e-mail (66%) and from the total of 304 TDR trainees (25%) who had initially been contacted. This illustrates the challenges to maintain contact with past trainees, as identified in previous evaluations of TDR’s capacity building activities [2]. In order to help keep track of former trainees, TDR launched the TDR Global initiative in 2016. The TDR Global platform, is an efficient and flexible web-based platform based on an existing open access “research networking tool”. It builds profiles of researchers affiliated to TDR and maps their expertise, their research activity and academic networks based mainly on their publications and co-authorship. The platform also helps track their career and professional achievements based on data they provide. This platform was launched publicly in November 2016 and data on its use and utility are being collected [12].

In addition, influence of the trainee’s selection on the training intervention outcome is difficult to assess. The current survey was not designed to analyse this element. Heads of institutions supported by TDR expressed in a previous survey [2] that TDR supported training had a high impact on the ability to develop research project. This, suggests that at least TDR supported training made a difference in some research skills.

The results presented in this paper highlight the important link perceived by respondents between TDR support and their career advancement. Most of the trainee respondents (80%) rated TDR support as a very important factor that influenced their professional career achievements. In order to address the potential social desirability bias (i.e. respondent giving a positive answer to please the questioner) a multiple choice questionnaire was included asking the importance of: (1) sponsoring organization; (2) the PhD supervisor/ mentor; and (3) the employer.

A high proportion of respondents (89%) remained in the field of research. The return rate to their region of origin (96%) is high with a very limited ‘brain drain’ rate to high-income countries (4%). These results do not take into account the 75% of trainees who could not be followed up. The TDR Global platform should potentially allow for a more comprehensive analysis. In the meantime, in order to assess the level of trainees who remained in the field of research, TDR developed a short survey on 212 trainees supported by TDR in Brazil. Brazil has a national research information system called Lattes which is coordinated by the Brazilian National Council for Scientific and Technological Development (CNPq). It is mandatory for researchers to fill in their profile on Lattes in order to apply for grants, faculty positions or staff appraisal. A search in the public interface of Lattes (http://lattes.cnpq.br/) showed that 86% of the 212 Brazilian TDR trainees had updated their profile in Lattes in the past two years and were still involved in research. Although Brazil is merely an illustrative example, this result reinforces the role of TDR on developing research capacity in low- and middle-income countries.

For decades, research capacity strengthening programmes targeting scientists in LMICs focused on north-south collaboration. According to the UNESCO Science Report 2015, from 2008 to 2014, the top three partners for the Economic Community of West African States (ECOWAS) came from France, the United States of America, and the United Kingdom, in that order [13, 14]. During this period, efforts increased research productivity in LMICs to a small extent. For example, in Sub-Saharan Africa the number of researchers rose from 0.9% to 1.1% (58 800 to 82 000) while in South Africa the number of researchers remained stable (0.3%). According to the same report, between 2008 and 2014, the percentage of worldwide scientific articles from Sub-Saharan Africa rose from 1.2 to 1.4 and from 0.5 to 0.7 in South Africa.

Some programmes have promoted a south-south collaboration approach to effectively address local health research problems and needs. An example is the Consortium for Advanced Research Training in Africa (CARTA) which is part of the African Institutions Initiative supported by the Wellcome Trust. CARTA aims to make a difference by rebuilding and strengthening the capacity of African universities to train locally skilled researchers [15]. A real time evaluation of the first four years of the CARTA programme [16] shows that although a critical mass of PhD and MSc graduates has been created, the long term impact, as for all the capacity building programmes, is still to be demonstrated. Indeed, although south-south collaboration should offer the possibility of facilitating the transfer of knowledge and best practices across the institutions [15], the effectiveness of this approach has to be carefully analysed [17]. The results presented in this article do not show any difference for a respondent from AFR, whether they studied in an LMIC or a HIC. This highlights the potential cost effectiveness of south-south collaboration. Collaboration across regions encourages mobility which is an important factor to develop independence following a post-doctoral position and gain leadership skills. Interestingly, most of the TDR trainee respondents worked in their own region during the period following their TDR grant. It would be important in a future study to analyse the factors involved in this low level of mobility and the level of south-south collaboration as well as north-south-south collaboration.

The survey also identified the challenges, bottlenecks and opportunities that trainees faced at various stages of their research careers. Although women are well represented in most WHO regions (except AFR), they do not always reach the same level and salary as men do. As a result of this survey, TDR initiated a new *Women in Science* programme to explore how to help more women enter and stay in science careers. Factors influencing access to TDR training grants from non-English speaking countries have not been identified properly and would need to be studied in future surveys.

The lessons learnt from this study are summarized below:

1. Retention of talent in countries and regions is an important factor for strengthening research capacity in an equitable and sustainable way. It needs to be taken into consideration in the programming of such activities so as to create incentives and facilitate the trainees’ return. Approaches such as a career development framework, re-entry grant schemes and working with both researchers and institutions in a coherent manner have shown to be important factors to limit ‘brain drain’. Targeted grant schemes should take into account the social situation of the grantee and set up specific criteria to encourage and support a scientific career pathway development within countries and internationally.
2. Further studies to better understand factors influencing the representation of women and their career development are needed. These would help develop capacity strengthening approaches to enhance gender equity. Responses to the following questions are essential in designing capacity strengthening programmes: how to enhance the engagement of women from AFR in research? What are the factors enhancing career development of women to leadership positions? Could lessons learnt in one region be helpful to another region?
3. In order to continue to build more effective research capacity in French and Portuguese speaking countries, further studies are needed to better understand the factors involved.
4. There are advantages in south-south collaboration i.e. trainees studying their degree in their own region. South-south collaborations are critical to foster the sustainability and impact of research capacity strengthening programmes. Collaboration between scientists and institutions in countries with more advanced health research capacities on one side, and their counterparts in less advanced health research capacity countries on the other side, are valuable. They strengthen the research capacity, support networking and information sharing, and also help retain scientists in low- and middle-income countries, limiting the ‘brain drain’ effect. However, at the same time there is a need to better understand the implications of low mobility on the opportunities to access leadership positions.
5. Training grant schemes are a good opportunity to promote multidisciplinary and intersectorial collaborations. They play an important role in achieving country plans and global development goals while providing a comprehensive and strategic perspective of issues and solutions, taking into account social, economic and environmental determinants. This would be helpful, especially in light of the Sustainable Development Goals (SDGs), where partnerships, multidisciplinary approaches and collaboration are considered essential to the achievement of the 2030 targets and therefore should be promoted and facilitated. There is a need to shift toward a multidisciplinary approach and intersectoral collaborations to reach the global development agenda for 2030 and ‘leave no one behind’
6. Grant schemes can also foster intersectoral mobility among academia, policy-makers, government agencies and the private sector, and decrease barriers to collaborative work. This should help address the identified needs for maintaining and enhancing efforts to bridge research, policy and practice.

The results of this study help highlight some factors influencing the effectiveness of TDR’s capacity strengthening programmes from 2000 to 2012. Lessons learnt could also help donors and policy-makers when setting programmes and funding priorities.

## Supporting information

S1 Checklist STROBE checklist. (DOC)
