# Supplementary material for: Tracking the career development of scientists in low- and middle-income countries trained through TDR’s research capacity strengthening programmes: Learning from monitoring and impact evaluation
Source: PLoS Negl Trop Dis. 2017 Dec 7;11(12):e0006112. doi: 10.1371/journal.pntd.0006112 (PMC5736233; doi:10.1371/journal.pntd.0006112)
Supplement: S1 Checklist — (DOC) [file pntd.0006112.s001.doc]

STROBE Statement—Checklist of items that should be included in reports of ***cohort studies***

|  | Item No | Recommendation |
| --- | --- | --- |
| **Title and abstract** | 1 | (*a*) Indicate the study’s design with a commonly used term in the title or the abstract  **Monitoring and evaluation is in the title** |
|  |  | (*b*) Provide in the abstract an informative and balanced summary of what was done and what was found  **See the abstract in the manuscript** |
| Introduction | | |
| Background/rationale | 2 | Explain the scientific background and rationale for the investigation being reported  **Regular external reviews of research capacity strengthening programmes helped organisations to evolve its strategy in light of the global environment so as to remain a fit-for-purpose programme. One recurrent recommendations from different evaluators was to better track the career development of grantees to help evaluate the influence of these early learning supports.**  **To respond to these recommendations a career tracking survey tool was developed to study the potential links between the grants received by TDR trainees and their career development.** |
| Objectives | 3 | State specific objectives, including any prespecified hypotheses  **The main objective was to identify factors that positively influenced and improved the research capacity and career development of TDR trainees** |
| Methods | | |
| Study design | 4 | Present key elements of study design early in the paper  **A questionnaire was designed on existing surveys of doctorate graduates conducted by the Organization for Economic Cooperation and Development (OECD), Eurostat, the European Commission Marie Sklodowska-Curie actions, Wellcome Trust, UNESCO and the US National Science Foundation. The range of topics covered by the survey included demographics, mobility (virtual, physical and sectoral), research outcomes, roles and responsibilities, competence development and skills utilization. Several drafts of the questionnaire were reviewed by five participating organizations and pre-tested in-house by ESF staff members, with the final questionnaire peer-reviewed by two independent international experts. The resulting questionnaire contained 52 questions, written in English were sent to previous TDR trainees.** |
| Setting | 5 | Describe the setting, locations, and relevant dates, including periods of recruitment, exposure, follow-up, and data collection  **The e-mail survey was launched with an explanatory cover note from ESF in September 2014 and was closed in November 2014** |
| Participants | 6 | (*a*) Give the eligibility criteria, and the sources and methods of selection of participants. Describe methods of follow-up  **TDR trainees who completed their doctorate or master’s degree between 2000 and 2012 with a TDR grant were identified in the TDR information and management system and included in this study These included recipients of any of the following scheme of grants: research training grants (RTG); re-entry grants (REG); the Multilateral Initiative on Malaria (MIM); research grants and institution strengthening grants (ISG).** |
|  |  | (*b*) For matched studies, give matching criteria and number of exposed and unexposed  **Not applicable** |
| Variables | 7 | Clearly define all outcomes, exposures, predictors, potential confounders, and effect modifiers. Give diagnostic criteria, if applicable  **The range of topics covered by the survey included demographics, mobility (virtual, physical and sectoral), research outcomes, roles and responsibilities, competence development and skills utilization.** |
| Data sources/ measurement | 8* | For each variable of interest, give sources of data and details of methods of assessment (measurement). Describe comparability of assessment methods if there is more than one group  **Survey by e-mails** |
| Bias | 9 | Describe any efforts to address potential sources of bias  **Respondents were asked to rate the importance of TDR support on achieving their professional career goals.Most of the trainee respondents (80%) rated TDR support as a very important factor that influenced their professional career achievements. In order to address the potential social desirability bias (i.e. respondent giving a positive answer to please the questioner) a multiple choice questionnaire was included asking the importance of: (1) sponsoring organization; (2) the PhD supervisor/ mentor; and (3) the employer.** |
| Study size | 10 | Explain how the study size was arrived at  **A total of 304 TDR trainees who completed their doctorate or master’s degree between 2000 and 2012 with a TDR grant were identified in the TDR information and management system. Trainees were contacted individually, through e-mail, to ascertain their willingness to participate in the career tracking survey and to update their personal information. From a total of 304 trainees identified, 117 trainees (39%) responded positively while 187 did not respond, either due to out of date e-mail addresses or possible lack of interest. Ultimately, 77 trainees responded to the survey (66% of those included).** |
| Quantitative variables | 11 | Explain how quantitative variables were handled in the analyses. If applicable, describe which groupings were chosen and why  **The number of respondents was logged on a daily basis and the percentage of responses on a weekly basis. A total of five reminders to participate in the survey were sent.** |
| Statistical methods | 12 | (*a*) Describe all statistical methods, including those used to control for confounding  **The survey data were imported into the Statistical Package for the Social Sciences (SPSS) for analysis** |
|  |  | (*b*) Describe any methods used to examine subgroups and interactions  **The survey was sent to five institutions participating in this survey . TDR results were disaggregated from this survey . Among the TDR responses, the data base was analysed for gender** |
|  |  | (*c*) Explain how missing data were addressed  **Since the survey was anonymous, there is no way to address the missing responses in the survey.** |
|  |  | (*d*) If applicable, explain how loss to follow-up was addressed  **Not applicable** |
|  |  | (*e*) Describe any sensitivity analyses  **Not applicable** |
| Results | | |
| Participants | 13* | (a) Report numbers of individuals at each stage of study—eg numbers potentially eligible, examined for eligibility, confirmed eligible, included in the study, completing follow-up, and analysed  **Among the 304 TDR trainees identified, 117 trainees expressed availability to participate and were included in the survey.. Ultimately, 77 trainees responded to the survey (66% of those included) and analysed.** |
|  |  | (b) Give reasons for non-participation at each stage  **From a total of 304 trainees identified, 117 trainees (39%) responded positively while 187 did not respond, either due to out of date e-mail addresses or possible lack of interest.**  **Response rate to a e-mail survey was quiet high (66%)** |
|  |  | (c) Consider use of a flow diagram  **This was not considered** |
| Descriptive data | 14* | (a) Give characteristics of study participants (eg demographic, clinical, social) and information on exposures and potential confounders  **These are given in the results section of the manuscript.** |
|  |  | (b) Indicate number of participants with missing data for each variable of interest  **The response rate for all the questions of the survey was between 95 and )7% except for a sensitive question on salary with a response rate of 62%** |
|  |  | (c) Summarise follow-up time (eg, average and total amount)  **Not applicable** |
| Outcome data | 15* | Report numbers of outcome events or summary measures over time  **The number of output i.e. data is 77 respondents** |
| Main results | 16 | (*a*) Give unadjusted estimates and, if applicable, confounder-adjusted estimates and their precision (eg, 95% confidence interval). Make clear which confounders were adjusted for and why they were included  **This is included in the manuscript in the results section** |
|  |  | (*b*) Report category boundaries when continuous variables were categorized  **Not applicable** |
|  |  | (*c*) If relevant, consider translating estimates of relative risk into absolute risk for a meaningful time period  **Not applicable** |
| Other analyses | 17 | Report other analyses done—eg analyses of subgroups and interactions, and sensitivity analyses  **None** |
| Discussion | | |
| Key results | 18 | Summarise key results with reference to study objectives  **In the discussion , we summarize the lesson learnt in six points** |
| Limitations | 19 | Discuss limitations of the study, taking into account sources of potential bias or imprecision. Discuss both direction and magnitude of any potential bias  **As mentioned in the discussion , the two main limitation factors have been identified i.e. the low number of participants ( 77) and the influence of trainee selection.** |
| Interpretation | 20 | Give a cautious overall interpretation of results considering objectives, limitations, multiplicity of analyses, results from similar studies, and other relevant evidence  **Given in the discussion part . See the discussion on the value of south-south collaboration compared to north south collaboration** |
| Generalisability | 21 | Discuss the generalisability (external validity) of the study results  **The results can be useful for any organisation willing to develop capacity strengthening programme with low and middle income countries. It will help also TDR to refine its portfolio of activities** |
| Other information | | |
| Funding | 22 | Give the source of funding and the role of the funders for the present study and, if applicable, for the original study on which the present article is based  **Funding by TDR** |

*Give information separately for exposed and unexposed groups.

**Note:** An Explanation and Elaboration article discusses each checklist item and gives methodological background and published examples of transparent reporting. The STROBE checklist is best used in conjunction with this article (freely available on the Web sites of PLoS Medicine at http://www.plosmedicine.org/, Annals of Internal Medicine at http://www.annals.org/, and Epidemiology at http://www.epidem.com/). Information on the STROBE Initiative is available at http://www.strobe-statement.org.
